# Autophagy-associated biomarkers ULK2, UVRAG, and miRNAs miR-21, miR-126, and miR-374: Prognostic significance in glioma patients

**DOI:** 10.1371/journal.pone.0311308

**Published:** 2024-09-30

**Authors:** Wajiha Amin, Syed Ather Enam, Sufiyan Sufiyan, Kulsoom Ghias, Mohammad Hamza Bajwa, Sahar Ilyas, Altaf Ali Laghari, Sana Naeem, Syed Hani Abidi, Nouman Mughal

**Affiliations:** 1 Department of Surgery, Aga Khan University Hospital, Karachi, Pakistan; 2 Center of Oncological Research in Surgery, Aga Khan University, Karachi, Pakistan; 3 Department of Biological & Biomedical Science, Aga Khan University Hospital, Karachi, Pakistan; 4 Department of Biomedical Sciences, Nazarbayev School of Medicine, Nazarbayev University, Astana, Kazakhstan; 5 Department of Life Sciences, Western Caspian University, Baku, Azerbaijan; Tabriz University of Medical Sciences, ISLAMIC REPUBLIC OF IRAN

## Abstract

As the pioneering study from Pakistan, our research distinctly focuses on validating the roles of autophagy-associated genes and MicroRNAs (miRs) in the unique context of our population for glioma prognosis. The study delves into the nuanced interplay of autophagy within a miR-modulated environment, prompting an exploration of its potential impact on glioma development and survival. Employing real-time PCR (qPCR), we meticulously assessed the expression profiles of autophagy genes and miRs in glioma tissues, complemented by immunohistochemistry on Formalin-fixed paraffin-embedded tissues from the same patients. Our comprehensive statistical analyses, including the data normality hypothesis Shapiro-Wilk test, the Mann-Whitney U-test, Spearman correlation test, and Kaplan-Meier survival analysis, were tailored to unravel the intricate associations specific to low- and high-grade glioma within our population. Clinicopathological analysis revealed a predominance of male patients (66%) with a median age of 35 years. Glioblastoma (32%) and Astrocytoma (36%) were the most prevalent histopathological subtypes. Molecular analysis showed significant correlations between prognostic markers (Ki-67, IDH-1, p53) and clinicopathological factors, including age, histological type, radiotherapy, and chemotherapy. In high-grade glioma, increased expression of AKT and miR-21, coupled with reduced ULK2 and LC3 expression was distinctly observed. While correlation analysis identified a strong positive correlation between ULK2 and UVRAG, PTEN, miR-7, and miR-100 in low-grade glioma, unveiling distinctive molecular signatures unique to our study. Furthermore, a moderate positive correlation emerged between ULK2 and mTOR, miR-7, miR-30, miR-100, miR-204, and miR-374, also between miR-21 and miR-126. Similarly, a positive correlation appeared between ULK2 and AKT, LC3, PI3K, PTEN, ULK1, VPS34, mTOR, Beclin1, UVRAG, miR-7 and miR-374. AKT positively correlated with LC3, PI3K, PTEN, ULK1, VPS34, mTOR, Beclin1, UVRAG, miR-7, miR-30, miR-204, miR-374, miR-126 and miR-21 weakly correlated with AKT and miR-30 in high-grade glioma, providing further insights into the autophagy pathway within our population. The enrichment analysis for miR-21, miR-126, and miR-374 showed MAPK pathway as a common pathway along with Ras, PI3K, and mTOR pathway. The low ULK2, UVRAG, and miR-374 expression group exhibited significantly poor overall survival in glioma, while miR-21 over-expression indicated a poor prognosis in glioma patients, validating it in our population. This study provides comprehensive insights into the molecular landscape of gliomas, highlighting the dysregulation of autophagy genes ULK2, and UVRAG and the associated miR-21, miR-126 and miR-374 as potential prognostic biomarkers and emphasizing their unique significance in shaping survival outcomes in gliomas within the specific context of the Pakistani population.

## Introduction

Gliomas, primary CNS tumors with significant molecular heterogeneity and a highly mutated genome constitute 50% of primary brain tumors, featuring a 12–15-month overall survival despite current medical interventions [1,2]. Recent studies identified dysregulated pathways in glioma, including PI3K/Akt/mTOR (cell survival), MAPK/ERK (cell growth), TP53 (cell cycle and DNA repair), RB pathway (cell cycle progression), IDH Mutation (metabolism and epigenetics), EGFR Amplification/Mutation (growth and therapy resistance), NF-κB pathway (inflammation and progression), and autophagy pathway (survival and therapy response) [3,4]. Recognizing these as crucial targets is imperative for effective treatment strategies, shaping both development and prognosis.

Autophagy, mediating the degradation of cytoplasmic components under stress conditions such as nutrient deprivation, hypoxia, endoplasmic reticulum (ER stress), cancer therapy, and pathogenic infection through the formation of autophagosomes [5] functions as a tumor suppressor during the initiation phase of cancer. Conversely, evasion of autophagy during cancer progression and therapeutic interventions contributes to a tumor-promoting effect [6]. The PI3K/Akt/mTOR signaling cascade, a critical component of the upstream autophagy pathway, is commonly dysregulated and is observed in 90% of glioblastoma (GBM) cases, and is caused by the overexpression of upstream activators, like epidermal growth factor receptor (EGFR) [7]. The exploration of autophagy mechanisms stands as a pivotal focus in understanding gliomagenesis and malignant transformation.

MicroRNAs (miRs), a class of non-coding RNAs, serve as crucial regulators of various cellular processes. In the context of cancer, miRs are frequently dysregulated in malignancies such as prostate, renal, colon, and glioblastoma, closely associated with cancer initiation, progression, and resistance to chemo-radiotherapy [8]. Particularly in glioma, autophagy-associated miRs have been implicated in contributing to chemoresistance, adding layer to their role in cancer biology [9]. Studies emphasizing the importance of miRs in autophagy regulation and the significance of autophagy progression in various cancer stages underscore the need for understanding the intricate interactions between autophagy and miRs in the context of glioma progression and patient survival. This understanding will contribute to elucidating the underlying tumor biology and potentially identifying novel therapeutic strategies. This study aims to determine and correlate the expression of autophagy markers, autophagy-associated signaling genes (PI3K/Akt/mTOR), and autophagy-associated miRs in both low and high-grade glioma.

In low-middle-income countries like Pakistan, that is experiencing a rise in cancer burden, understanding the molecular nuances is vital. Challenges, such as health costs and follow-up issues, underscore the need for specific insights tailored to these regions [10]. Deciphering the role of autophagy and related microRNAs provides a comprehensive view of molecular dynamics. Validation studies in our population could contribute globally and hold promise for identifying biomarkers, crucial for improved diagnostics and targeted treatments. Correlating molecular characteristics with overall survival in the understudied Pakistani population is essential for advancing cancer research both locally and globally.

## Methods

### Patient selection

For this study, 50 fresh glioma tumor samples, divided as low-grade glioma (LGG; Grade II; N = 15) and high-grade glioma (HGG; Grade III, Grade IV; N = 35) and 50 matching formalin-fixed paraffin-embedded (FFPE) blocks from patients, who had a confirmed diagnosis of glioma on histology and CT (Computerized Tomography)/ MRI (Magnetic Resonance Imaging), and underwent surgery at the Aga Khan University Hospital, Karachi between 21^st^ September 2019–11 April, 2021 were collected and survival follow-up check was done until 2022. Patient details, such as therapeutic data (chemotherapy, radiotherapy), histopathology, tumor grade, and molecular marker (Ki-67) were retrieved from hospital medical records. This study was approved by the ethical review committee of Aga Khan University (AKU-ERC # 2019-1945-5110 with an extension AKU-ERC # 2021-1945-17282). All eligible participants involved in the study provided written informed consent for the utilization of their tissue samples.

### Histopathology and immunohistochemistry

Histological sections of tumor tissues FFPE were examined and graded by a histopathologist at Aga Khan University. In each FFPE sample, IHC-based expressions of ATRX, p53, IDH1-R132H, LC3-II were examined. Briefly, after deparaffinization in xylene and rehydrated in serial graded alcohol, antigen retrieval was conducted using retrieval solution (EnVision + system Dako, Denmark) with the dilution of 1:50 with distilled water in a coplin jar placed in preheated (90–95°C) water bath for 40 minutes, followed by cooling at room temperature. Following a 10-minute blocking of endogenous peroxidase activity with (0.03% hydrogen peroxide containing sodium azide, Dako, Denmark). Mouse monoclonal antibodies targeting IDH-1 R132H (clone: H09, Dianova), ATRX (clone: BSB-108), and p53 (DO7, Cell Marque), as well as a rabbit polyclonal antibody against LC3-II (ab51520), were administered. The incubation was done for 30 minutes in a humidity chamber at room temperature. Subsequently, the samples were washed three times using phosphate buffer saline (PBS) buffer with a pH of 7.6 with each wash lasting for a duration of 2 minutes. After that, biotinylated secondary antibodies were applied, followed by a washing step using a PBS solution. Subsequently, the samples were incubated with horseradish peroxidase (HRP) labelled polymer-HRP anti-mouse Dako, Denmark for a duration of 30 minutes, followed by another round of washing as previously described. The chromogen 3,3-diaminobenzidine (DAB) was applied for 1 minute as per the manufacturer’s protocol. The slides were counterstained with hematoxylin, followed by dehydration in ethanol and subsequently with xylene. Mounting of slides were done using Dako Toluene free Mounting Medium (code CS705). Negative controls were prepared by substituting the primary antibody with PBS, while brain tumor samples previously characterized for over-expression of IHC markers were used as positive controls. For the IHC assessment, all prepared slides were examined using Olympus BX43 light microscope at 20X objective magnification. Photographs were obtained from PC-driven digital camera (Olympus) and analyzed using CAPTURE infinity imaging solution tool. IDH-1, ATRX and p53 were scored as positive or negative. LC3-II punctae were quantified using four-tiered scale: score 1, punctae ≤ 10 per cells; score 2, punctae 11–20 per cells; score 3, punctae >20 per cells [11].

### RNA extraction, DNase treatment of RNA, and cDNA synthesis

Total RNA was extracted from glioma tissue samples using TRIzol^®^-chloroform (Invitrogen; Thermo Fisher Scientific, Inc; Cat # 15596018) method, following manufacturer’s instructions. RNA concentration and purity was assessed using Nanodrop^®^ 2000c spectrophotometer (Thermo Scientific, Waltham, MA, USA). The RNA samples were stored at -80°C for further use.

Prior to cDNA conversion, each RNA sample was treated with DNase-I (RNase-free) 1U/1μl (Thermo Fisher Scientific, Cat. No. EN0521). For this, 1μg of the total RNA template was pooled in a 200 μl tube with 1ul of DNase-I, a 10X reaction buffer comprising MgCl_2_ and nuclease-free water for a final volume of up to 10 μl. The reaction was incubated at 37°C for 30 minutes followed by addition of 1μl of 50 Mm EDTA, and incubation at 65°C for 10 minutes. The treated RNA was stored at -80°C. The treated RNA samples were converted to cDNA using RevertAid first strand cDNA synthesis kit (Thermo Fisher Scientific, Cat. No. K1612). In the first step, 1 μl of treated RNA was mixed with 1 μl of 10 μM Random primers, and 10 ul of nuclease-free water in 200 μl tube. The tube was incubated for 5 minutes at 65°C, briefly centrifuged and incubated on ice for 1 minute. The reaction mixture was further supplied with 4 μl of 5X reaction buffer, 1μl of Ribonuclease inhibitor, 2 μl of 10 μM dNTPs mix each and 1 μl of RevertAid reverse transcriptase enzyme and incubated at following conditions: 50°C for 5 minutes, then 64°C for 60 minutes, and finally 70°C, for 5 minutes. Additionally, the prepared cDNA was diluted by the addition of 180 μl nuclease-free water to make up the reaction volume 200 μl. The prepared cDNA samples were stored at -20°C until further use.

### Analysis of gene expression using quantitative real-time PCR (qPCR)

Prevailing data in the literature (Table 1) indicated that deregulation of certain miRs expression contributes to the mechanism of cancer formation. Therefore, considering the importance of autophagy-regulating miRs, each sample was subjected to a panel of 7 autophagy-related miRs. The expression of autophagy genes, LC3, ULK1/2, Beclin1, UVRAG, and autophagy-associated signaling pathway genes, PI3K, AKT, mTOR, PTEN and autophagy-related miRs (miR-7, miR-21, miR-30, miR-100, miR-126 and miR-374) were determined through the quantitative real-time PCR (qPCR) using QIAGEN’S Rotor-Gene Q machine. A 10 μl reaction mixture was set by combining 2 μl of cDNA template with 5 μl of PowerUp^™^ SYBR^™^ Green Master Mix (Thermo Fisher Scientific, Cat. No. A25742), 1 μl forward and reverse primers (10 μM) each (S1 Table) (Eurofins, USA), and 2 μl of nuclease free water. The above reactions were subjected to the following thermal cycling conditions; initial hold for 2 mins at 50°C, another hold for 2 minutes at 95°C followed by 40 cycles for denaturation for 15 sec at 95°C, annealing for 60 sec at 60°C. A melt curve analysis was set up between 50°C to 90°C with an increment of 1°C at each step to plot the specificity of the products. Each reaction was run in duplicate with a non-template and non-primer as an experimental control. A β-Actin gene and small nuclear RNA-U6 were used as an endogenous control. A relative fold change in gene and miR expression in each sample was calculated using ΔΔCt (ΔCt of genes/ miRs–Average ΔCt of reference group (Low Grade)) Livak 2^-ΔΔCt^ method [12].

**Table 1:** List of autophagy-related genes and target miRs from literature.

miRs	Target genes	Type of Target Interaction	References
**miR-7**	PI3K. mTOR	Direct	[13]
**miR-30**	BECN1	Direct	[14,15]
**miR-100**	mTOR	Direct	[16]
**miR-126-3p**	AKT	Direct	[17]
**miR-204**	LC3	Direct	[18]
**miR-374**	UVRAG	Direct	[19]
**miR-21**	PTEN	Direct	[20]

### Statistical analysis

Shapiro-wilk with Maan-Whitney tests were applied to determine the difference in gene expression between in the LGG and HGG groups. In the next step, we applied Spearman’s rank correlation coefficient test to correlate expression of different genes in LGG and HGG groups using the following log scale 0.9 to 1 (-0.9 to -1) very strong positive (negative) correlation; 0.7 to 0.9 (-0.7 to -0.9) high positive (negative) correlation; 0.5 to 0.7 (-0.5 to -0.7) moderate positive (negative) correlation; 0.3 to 0.5 (-0.0 to -0.5) low positive (negative) correlation; 0.0 to 0.3 (-0.0 to -0.3) negligible correlation [21]. Similarly, Pearson Chi-square was applied for the demographic analysis with clinical parameters. R statistical platform base software version 3.3.2 and R-studio version. Cherry Blossom (3c53477a, 2023-03-09) was used for statistical data analysis and graphical representation. In all analyses, a p-value less than 0.05 was considered statistically significant.

For survival analysis, patients were divided into low and high expression groups, using median expression of genes and miRs as the cut-off value, and a log-rank test was applied for survival analysis and Kaplan-Meier survival plots were generated. In addition, Cox regression analysis was performed on the same group of patients to quantify the comparative hazard. Receiver operating characteristics (ROC) curves were generated to test the predictive value of the autophagy genes and miRs signature for overall survival using R package (survival ROC). For functional enrichment analysis we used Metascape to explore the biological processes associated with these gene groups. We utilized KEGG pathways, WikiPathways, and oncogenic signature gene set enrichment analysis, with an adjusted P-value cutoff of < 0.05.

## Results

### Clinical characteristics of the study population

A total of 50 glioma patients were recruited in our study, out of which, 17 (34%) were females and 33 (66%) were males, with a median age of 35 years. The most prevalent histopathology groups were Astrocytoma 18 (36%) and glioblastoma 16 (32%) followed by oligodendroglioma 6 (12%). Twenty patients (40%) had postoperative Karnofsky Performance Scale KPS scores of less than 80, while 22 (44%) of the patients had KPS scores above 80. The average life expectancy in our study group was 16.8 months, ranging from 0.4 to 33.8 months (Table 2).

**Table 2:** Summary of clinicopathological characteristics of glioma cases.

Clinicopathological features	Values (%)
**Gender**	
Male	33 (66%)
Female	17 (34%)
**Age (years)**	
Medians (range)	35
**Histological type (WHO CNS5 2021 grade)**	
2	15 (30%)
3	5 (10%)
4	30 (60%)
**Histological grades**	
LGG	15 (30%)
HGG	35 (70%)
**Histological groups**	
Astrocytoma	18 (36%)
Glioblastoma	16 (32%)
Oligodendroglioma	6 (12%)
High grade glioma with IDH-WT	4 (8%)
Low grade glioma with IDH-WT	6 (12%)
**Status at 4 years**	
Dead	23 (46%)
Alive	26 (52%)
LTFU	1 (20%)
**Recurrence**	
No recurrence	43 (86%)
Recurrence	7 (14%)
**Radiotherapy**	
Yes	31 (64.58%)
No	17 (35.41%)
**Chemotherapy**	
Yes	34 (70.83%)
No	14 (29.16%)
**Adjuvant chemoradiotherapy**	31 (42%)
**Postoperative KPS score**	
> = 80	22 (44%)
<80	20 (40%)
**Overall survival months Median (Range)**	16.8 (0.4–33.8)
**HGG**	11.86
**LGG**	28.2

Pearson chi-square correlation analysis showed that the prognostic marker Ki-67 was significantly correlated with age, histological type, radiotherapy, tumor grade, and tumor current status (p<0.005). Moreover, IDH-1 showed a significant correlation with age and current status and p53 with chemotherapy. IDH1 and p53 both were significantly correlated with histological types (p < 0.05) (Table 3).

**Table 3:** Correlation of molecular protein expression ATRX, IDH1, p53 and Ki67 with clinicopathological factors. Rows with variables exhibiting significant correlation (p<0.05) are shaded grey.

Factors	Total Samples (n = 50)	ATRX			IDH1			p53			Ki67		
		Retained n = 43	Loss n = 7	p-value	Wild Type n = 31	Mutant n = 19	P-value	Mutant n = 33	Wild Type n = 17	P-value	High n = 36	Low n = 14	P-value
	**Age group**												
<40	32	26 (81.2%)	6 (18.75%)	p = 0.2	16 (50%)	16 (50%)	**p = 0.033**	23 (71.87%)	9 (28.12%)	p = 0.3	19 (59.37%)	13 (40.62)	**p = 0.009**
>40	18	17 (94.44%)	1 (5.55%)		15 (83.33%)	3 (16.66%)		10 (55.5%)	8 (44.4%)		17 (94.44%	1 (5.55%)	
	**Gender**												
Female	17	16 (94.11%)	1 (5.88%)	p = 0.3	13 (76.47%)	4 (23.52%)	p = 0.21	9 (52.94%)	8 (47.05%)	p = 0.2	14 (82.35%)	3 (17.64%)	p = 0.32
Male	33	27 (81.81%)	6 (18.8%)		18 (54.54%)	15 (44.44%)		24 (72.72%)	9 (27.27%)		22 (66.66%)	11 (33.33%)	
	**Grades**												
LGG (grade II)	15	12 (80%)	3 (20%)	p = 0.6	8 (53.33%)	7 (46.66%)	p = 0.52	10 (66.66%)	5 (33.33%)	p = 1	1 (6.66)	14 (93.3%)	**p = 0.00**
HGG (III, IV)	35	31 (88.57%)	4 (11.47%)		23 (65.71%)	12 (34.28%)		23 (56.71%)	12 (34.28%)		35 (100%)	0 (0%)	
	**Chemotherapy**												
Yes	34	29 (85.29%)	5 (14.70%)	p = 1	21 (61.76%)	13 (38.23%)	p = 1	26 (76.47%)	8 (23.52%)	**p = 0.004**	27 (79.41)	7 (20.58%)	p = 0.7
No	14	12 (85.71%)	2 (14.28%)		8(57.14%)	6 (42.85)		6 (42.85%)	8 (57.14%)		7 (50%)	7 (50%)	
	**Radiotherapy**												
Yes	31	27 (87.09%)	4 (12.90%)	p = 0.6	18 (58.06%)	3 (9.67%)	p = 0.7	23 (74.19%)	8 (25.80%)	p = 0.2	25 (80.64)	6 (19.35%)	**p = 0.05**
No	17	14 (82.35%)	3 (17.64%)		11 (64.70%)	6 (35.29%)		9 (52.94%)	8 (47.05%)		9 (52.94%)	8 (47.05%)	
	**Recurrence**												
Yes	7	6 (85.71%)	1 (14.28%)	p = 1	3 (42.85%)	4 (57.14%)	p = 04	5 (71.42%)	2 (28.57%)	p = 1	4 (57.15%)	3 (42.85%)	p = 038
No	43	37 (86.04%)	6 (13.95%)		28 (65.11%)	15(34.88%)		28 (65.11%)	15 (34.88%)		32 (74.44%)	11 (25.58%)	
	**Current Status**												
Dead	23	21 (91.30%)	2 (8.69%)	p = 0.5	20 (86.95%)	3 (13.04%)	p = 0002	13 (56.52%)	10 (43.47%)	p = 0.1	22 (95.65%)	1 (4.34%)	**p = 0.001**
Alive	26	21 (80.76%)	5 (19.23%)		10 (38.46%)	16 (61.53%)		20 (76.92%)	6 (23.07%)		13 (50%)	13 (50%)	
	**Histological Types**												
Oligodendrogioma	6	6 (100%)	0 (0%)	p = 0.8	0 (0%)	6 (100%)	**p <0.001**	3 (50%)	3 (50%)	p = 0.1	4 (66.6%)	2 (33.3%)	**p = 0.001**
Glioblastoma	16	14 (87.5%)	2 (12.5%)		16 (100%)	0 (0%)		8 (50%)	8 (50%)		16 (100%)	0 (0%)	
Astrocytoma	18	15 (83.33%)	3 (16.66%)		5 (27.77%)	13 (72.22)		16 (88.8%)	2 (11.11%)		11 (61.1%)	7 (38.8%)	
High grade glioma	4	3 (75%)	1 (25%)		4 (100%)	0 (0%)		2 (50%)	2 (50%)		4 (100%)	0 (0%)	
Low grade glioma	6	5 (83.33%)	1 (16.66%)		6 (100%)	0 (0%)		4 (66.6%)	2 (33.3%)		1 (16.6%)	5 (83.3%)	

### Expression of autophagy marker *LC3-II* in LGG and HGG group

**Fig 1:**
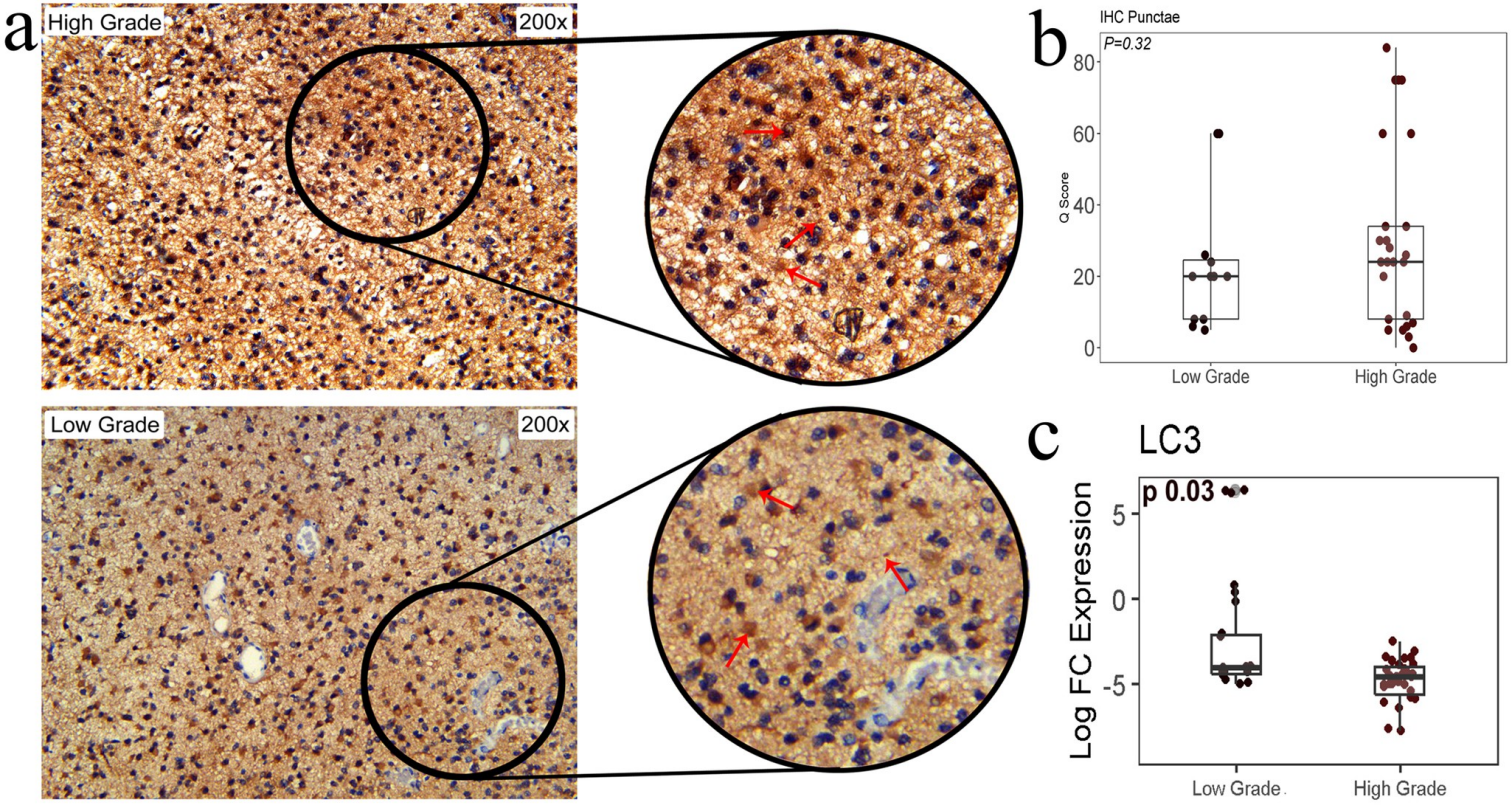
LC3-II in low- and high-grade glioma. (a). Immunohistochemistry images. (b). LC3-II in low-grade and high-grade Glioma. (c). Quantification for LC3-II punctea was based on the intensity score from 1–3, where score 1, punctae ≤ 10 per cells; score 2, punctae 11–20 per cells; score 3, punctae >20 per cells c) RT-qPCR shows a significant difference in median expression of LC3-II in low and high-grade glioma tissues using Mann-Whitney test.

Utilizing IHC, we investigated the expression of LC3-II to establish autophagic flux in patient biopsy tissues. Among the 50 cases examined, 49 displayed cytoplasmic LC3-II puncta in various cellular regions. The median Q scores for LC3-II were 20 and 24 for LGG and HGG, respectively. Interestingly, the difference in cytoplasmic expression of LC3-II between LGG and HGG was not found to be statistically significant (*p* = 0.32) (Fig 1a and 1b). Next, we examined the differential mRNA expression of LC3-II in both LGG and HGG samples. The qPCR analysis revealed a statistically significant difference in LC3-II mRNA expression, with HGG samples showing a -4.09-log fold lower expression compared to LGG (p = 0.03) (Fig 1c).

### Analysis of autophagy-associated genes in LGG and HGG group

**Fig 2:**
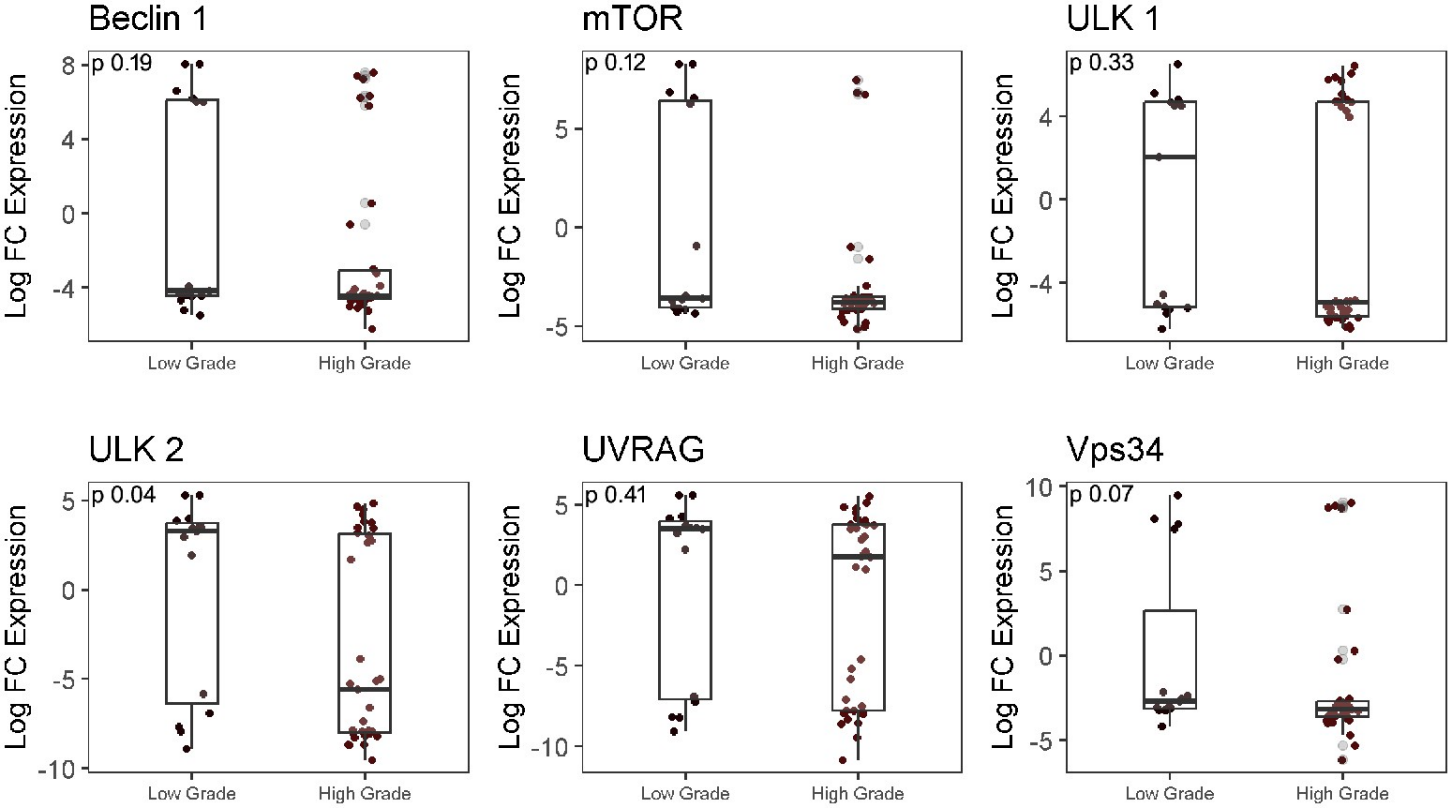
RT-qPCR analysis for Beclin1, mTOR, ULK1, ULK2, UVRAG, Vps34 genes expression in LGG and HGG group. Log fold difference expression of genes between LGG and HGG was determined using Mann-Whitney test.

To further explore the differential expression of autophagy-associated genes in LGG and HGG groups, the expression of seven genes related to the core autophagy activation pathway, namely ULK1 complexes (ULK1, ULK2), Beclin1/PI3K complex interacting proteins (Beclin1, UVRAG, Vps34), and ATG-8 ubiquitin-like conjugation system (LC3B) was investigated. No significant difference in expression of the ULK1 gene was observed between LGG and HGG samples (p = 0.33), while analysis of ULK2 showed a significant 3.09 log fold higher expression in LGG as compared to HGG (p = 0.04). VPS34, Beclin1, UVRAG, and mTOR did not exhibit a significant difference (p>0.05) in expression between the two groups (Fig 2).

### Analysis of PTEN/PI3K/AKT pathway genes in LGG and HGG group

**Fig 3:**
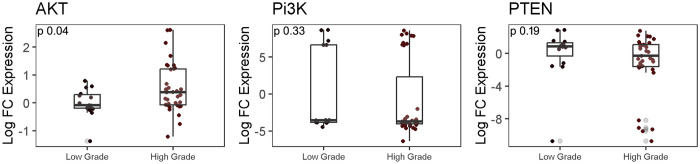
RT-qPCR analysis for pathway associated genes Pi3K/PTEN/AKT gene expression in LGG and HGG group. Log fold difference expression of genes between LGG and HGG was determined using Mann-Whitney test.

Next, the differential expression of PTEN/PI3K/AKT genes was examined, which are components of the upstream pathway. The mRNA expression of the AKT gene was found to be 0.5 log fold higher (p = 0.04) in HGG as compared to LGG group. The expression of PI3K and PTEN, however, were comparable between the two groups (p = 0.19) (Fig 3).

### Analysis of differential expression of autophagy-associated miRNAs in LGG and HGG group

**Fig 4:**
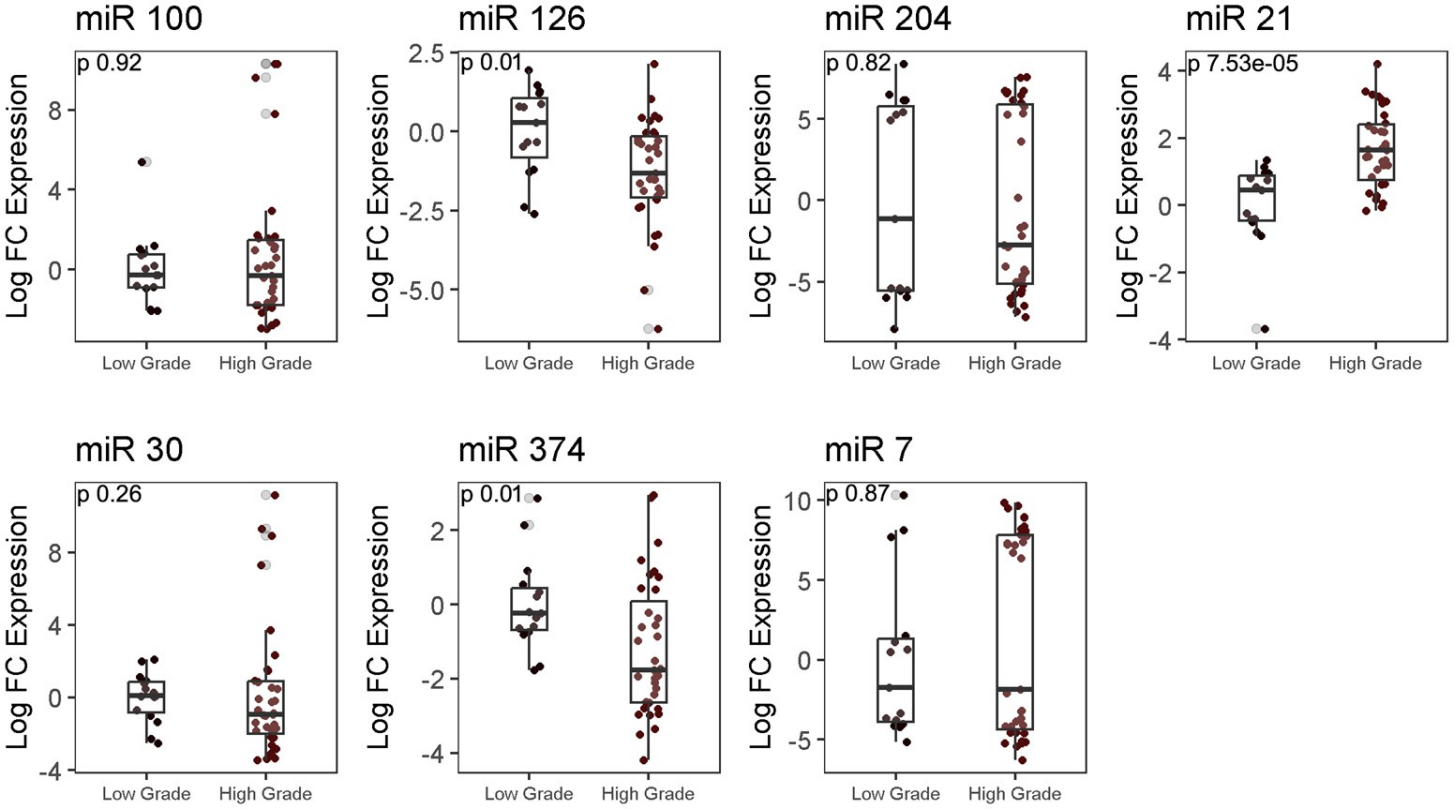
RT-qPCR analysis for micro-RNA expression in LGG and HGG group. Log fold difference expression of microRNA between LGG and HGG was determined using Mann-Whitney test.

Analysis of the expression of autophagy-associated miRs showed the expression of miR-126 and miR-374 to be 1.17-fold (p = 0.01) and 1.28-fold (p = 0.01) higher in LGG as compared to HGG group, while miR-21 expression was found to be 1.76- fold more in HGG as compared to LGG (p = 0.000075). Expressions of other tested miRs miR-7, miR-30, miR-100, miR-204 were comparable between the two groups (Fig 4).

### Correlation of expression of miRs and other autophagy-associated genes in LGG and HGG group

The Spearman’s rank correlation coefficient test was used to correlate expression of different genes in LGG and HGG groups. In the LGG group, a significant strong positive correlation was observed between *UKL2* and *miR-7* (rs = 0.85), ULK2 and miR-100 (rs = 0.81). Similarly, a moderate positive correlation was observed between ULK2 and miR-30 (rs = 0.56), ULK2 and miR-204 (rs = 0.58), ULK2 and miR-374 (rs = 0.68), LC3 and miR-374 (rs = 0.66). Likewise, miR-21 exhibited a moderate correlation between miR-126 (rs = 0.56).

Similarly, a strong positive correlation was observed between ULK2 and PTEN (rs = 0.91), ULK2 and UVRAG (rs = 0.80) while a moderate correlation between ULK2 and mTOR (rs = 0.59) (Table 4).

**Table 4:** Analysis of correlation between the expression of different genes in the LGG group. The correlation was determined using Spearman’s correlation test. Each column shows the coefficient of correlation (rs). Correlations with significant p-values (p<0.05) are shown in bold.

Correlation between Gene to miRs in Low Grade Glioma							
Genes/miRs	miR-7	miR-30	miR-100	miR-126	miR-204	miR-374	miR-21
**ULK2**	**0.85**	**0.56**	**0.81**	0.15	**0.58**	**0.68**	0.21
**AKT**	**0.45**	0.19	0.04	0.10	0.19	0.16	0.03
**LC3**	0.24	0.25	0.34	-0.16	0.25	**0.66**	-0.27
**miR-21**	0.12	0.05	0.01	**0.56**	0.23	0.08	1.00

Correlation between Gene to Genes in Low Grade Glioma										
Genes/miRs	ULK2	AKT	LC3	PI3K	PTEN	ULK1	Vps34	mTOR	Beclin	UVRAG
**ULK2**	1.00	0.39	0.39	**0.45**	**0.91**	0.41	**0.51**	**0.59**	**0.51**	**0.80**
**AKT**	0.39	1.00	0.18	-0.03	0.24	-0.31	-0.21	-0.05	0.11	0.30
**LC3**	0.39	0.18	1.00	-0.01	0.38	0.33	0.11	0.39	0.10	0.26
**miR-21**	0.21	0.03	-0.27	0.13	0.31	0.04	0.38	0.30	0.13	0.25

In the HGG group, a significant low positive correlation was observed between ULK2 and miR-7 (rs = 0.40), ULK2 and miR-374 (rs = 0.50). Likewise, AKT was moderately correlated with miR-7 (rs = 0.66) and showed a low positive correlation between AKT and miR-30 (rs = 0.41), miR-204 (rs = 0.40), miR-374 (rs = 0.35), miR-21 (rs = 0.38) respectively. Also, miR-30 has a positive correlation with miR-21 (rs = 0.40).

Similarly, AKT correlated positively with UVRAG (rs = 0.69), VPS34 (rs = 0.56), ULK1 (rs = 0.59), ULK2 (rs = 0.51), PTEN (rs = 0.48), and PI3K (rs = 0.48). ULK2 correlated with ULK1 (rs = 0.53), PTEN (rs = 0.63) PI3K (rs = 0.49), VPS34 (rs = 0.64), mTOR (rs = 0.59), Beclin (rs = 0.55), UVRAG (rs = 0.66) and AKT (rs = 0.51). A weak positive correlation of miR-21 was observed with AKT (rs = 0.38) and LC3 correlated positively with PTEN (rs = 0.47), ULK1(rs = 0.45), VPS34 (rs = 0.48) in high-grade glioma (Table 5).

**Table 5:** Analysis of correlation between the expression of different genes in HGG group. Correlation was determined using Spearman’s correlation test. Each column shows the coefficient of correlation (rs). Correlations with significant p-values (p<0.05) are shown in bold.

Correlation between Gene to miRs in High Grade Glioma							
Genes/miRs	miR-7	miR-30	miR-100	miR-126	miR-204	miR-374	miR-21
**ULK2**	**0.40**	0.27	0.17	0.32	0.13	**0.50**	0.21
**AKT**	**0.66**	**0.41**	0.30	**0.38**	**0.40**	**0.35**	**0.38**
**LC3**	0.19	**0.35**	0.03	0.15	-0.02	0.26	-0.01
**miR-21**	0.26	**0.40**	0.12	0.08	-0.14	0.11	1.00

Correlation between Gene to Genes in High Grade Glioma										
Genes/miRs	ULK2	AKT	LC3	PI3K	PTEN	ULK1	Vps34	mTOR	Beclin1	UVRAG
**ULK2**	1.00	**0.51**	**0.33**	**0.49**	**0.63**	**0.53**	**0.64**	**0.59**	**0.55**	**0.66**
**AKT**	**0.51**	1.00	**0.19**	**0.48**	**0.48**	**0.59**	**0.56**	**0.43**	**0.34**	**0.69**
**LC3**	0.33	0.19	1.00	0.30	0.47	0.45	0.48	0.33	0.27	0.26
**miR-21**	0.21	**0.38**	-0.01	0.12	0.03	0.24	0.09	-0.05	-0.14	0.29

### Association of expression of autophagy-associated genes and miRs with patient’s survival and cancer prognosis

Considering that AKT, ULK2, LC3, and miR-21 expressions were significantly correlated with glioma grade, we hypothesized that these genes might affect the survival/cancer prognosis of the patients. To confirm this possibility, the patients were divided into high AKT, ULK2, LC3, and miR-21 expression, and low expression groups. Cox regression was performed on the two groups and a high-expression group was used as a reference. The hazard ratios (HR) >1 and <1 was used as low and high hazard risk indicators, respectively. Univariate Cox regression revealed that low ULK2 (HR, 2.54; p = 0.03) and low UVRAG (HR, 2.78; p = 0.02) low miR-374 (HR, 2.568; p = 0.03) and high miR-21 (HR, 2.16; p = 0.07) were associated with poor overall survival and multivariate cox regression confirmed that miR-21 (HR, 2.62; p = 0.04) was an independent predictor for worse overall survival (Table 6).

**Table 6:** Univariate and multivariate cox proportional hazard analysis of autophagy-associated genes and miRs for overall survival OS.

	**Univariate Cox Regression**			
**Variable**	**Reference group**	**HR**	**P-value**	**CI**
**ULK2**	High ULK2 expression group	2.544	**0.034**	1.074–6.029
**UVRAG**	High UVRAG expression group	2.789	**0.020**	1.174–6.622
**miR-374**	High miR-374 expression group	2.568	**0.032**	1.085–6.077
**miR-21**	Low miR-21 expression group	2.168	0.078	0.195–1.089
	**Multivariate Cox Regression**			
**Variable**	**Reference group**	**HR**	**P-value**	**CI**
**ULK2**	High ULK2 expression group	1.967	0.149	0.784–4.935
**UVRAG**	High UVRAG expression group	2.278	0.124	0.797–6.509
**miR-21**	Low miR-21 expression group	2.626	**0.041**	0.151–0.962
**miR-374**	High miR-374 expression group	1.404	0.497	0.528–3.733

Abbreviation: HR = Hazard ratio Patients classified into low and high expression group according to median value of each miR and gene and high expression group was considered as a reference group *p<0.05.

**Fig 5:**
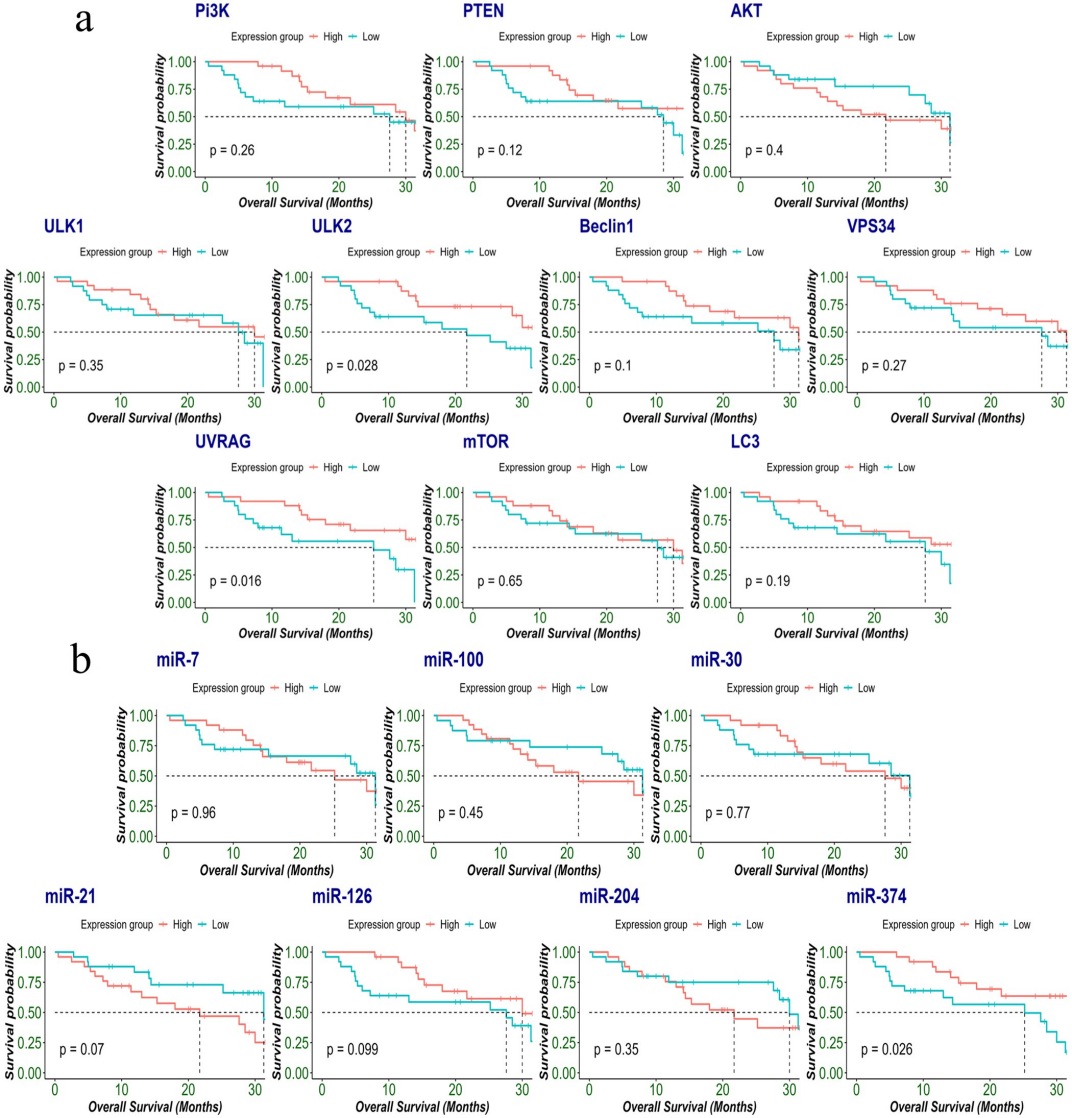
(a). The prognosis of autophagy genes and (b). miRs in glioma patient tissue samples (n = 50). Kaplan-Meier survival analysis was performed after Glioma patients were clinically followed up for 40 months post-surgery and segregating patients into two groups based on median expression: Patients with high expression of genes (red) and patients with low expression of genes (blue).

In the next step, the above predictors ULK2, UVRAG, miR-21 and miR-374 along with significantly expressed genes AKT, ULK2, LC3 and miRs miR-21, miR-126 and miR-374 were included for survival analysis. The results indicated that low expression of ULK2, and UVRAG were found to be statistically correlated with poor overall survival (log-rank: 0.028, 0.016, 0.05, respectively) (Fig 5a). Furthermore, low miR-374 was significantly correlated with poor overall survival OS in glioma patients (log-rank: 0.033) Increased miR-21 expression levels were also predictive of reduced overall survival (log-rank: 0.07) (Fig 5b). Moreover, areas under the receiver operating characteristics curves (AUCs) for UVRAG, ULK2 and miR-374 were 0.893, 0.702 and 0.709 respectively (S1 Fig). We also looked at the existing molecular markers of glioma, and the AUC was 0.676 for IDH, 0.603 for p53 and 0.543 for ATRX. The result showed that UVRAG, ULK2 and miR-374 had better risk prediction capabilities than IDH, P53 and ATRX in the study group (S2 Fig).

**Fig 6:**
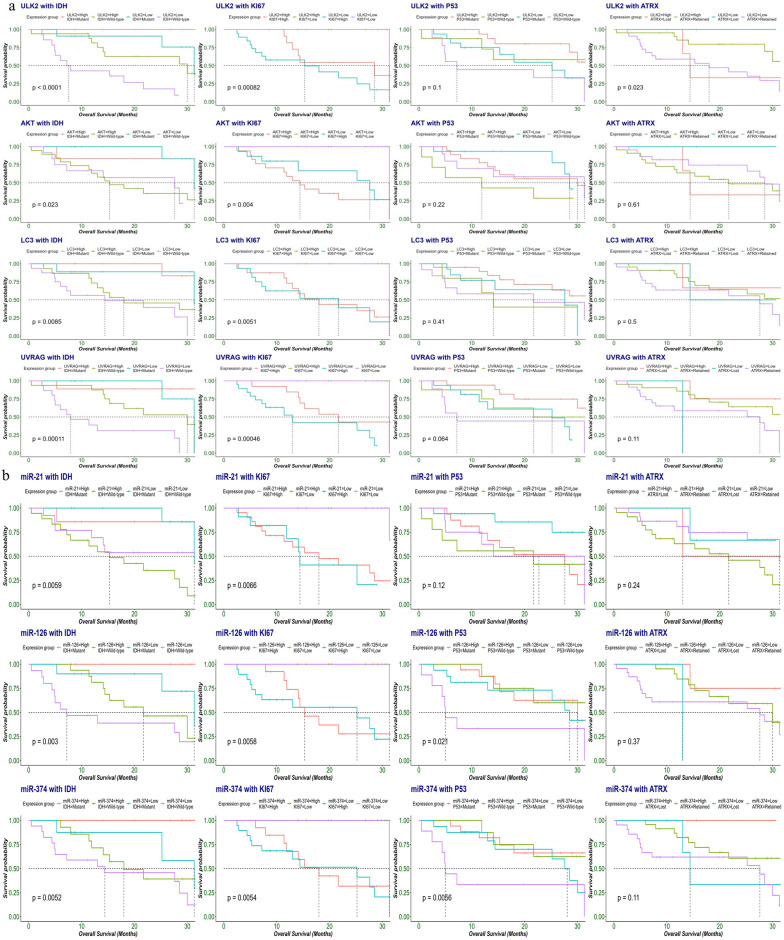
The prognosis of autophagy genes (a) and miRs (b) in background of IDH1, Ki67 and p53 genes in glioma patient tissue samples (n = 50). Kaplan-Meier survival analysis was performed after glioma patients were clinically followed up for 40 months post-surgery. Molecular analysis of genes and miRs (were, ULK2, LC3, miR-21, miR-126 and miR-374) transcription levels in Glioma tissue was measured by RT-qPCR, while IDH1, Ki67 and p53 protein expression in paraffin-embedded glioma tissue sections was detected using immunohistochemistry.

High and low expression groups were further classified based on the molecular marker status. Kaplan-Meier plots showed that patients with a low ULK2 expression, wild-type IDH, high Ki67 and retained ATRX expression had significantly poor OS (log-rank: <0.0001) (log-rank: 0.00082) (log-rank: 0.023) respectively. Interestingly, patients with IDH-wild type showed a significant trend towards poor OS in both low and high LC3 expression groups (log-rank: 0.066). Similarly, the IDH wild-type and high Ki67 expression groups were significantly associated with poor OS when compared with the low LC3 expression group (log-rank: 0.0085), (log-rank: 0.005) respectively and the Low UVRAG expression groups (log-rank: 0.00011), (log-rank: 0.0046) respectively. High miR-21 expression and low miR-126 and miR-374 expression correlated with poor overall survival in IDH-wildtype tumors (log-rank: 0.0059) (log-rank: 0.003) (log-rank: 0.0052) respectively. The group with high ki67 expression showed significantly poor overall survival when considered independently of miR-21, miR-126 and miR-374 expression levels (log-rank: 0.0066) (log-rank: 0.0058) (log-rank: 0.0054). Also, low miR-126 and low-374 expression level was significantly associated with poor OS in p53 wildtype tumor patients (log-rank: 0.021) (log-rank: 0.0056) respectively (Fig 6a and 6b).

### Functional and pathway enrichment analysis of miRNA target genes using metascape

**Fig 7:**
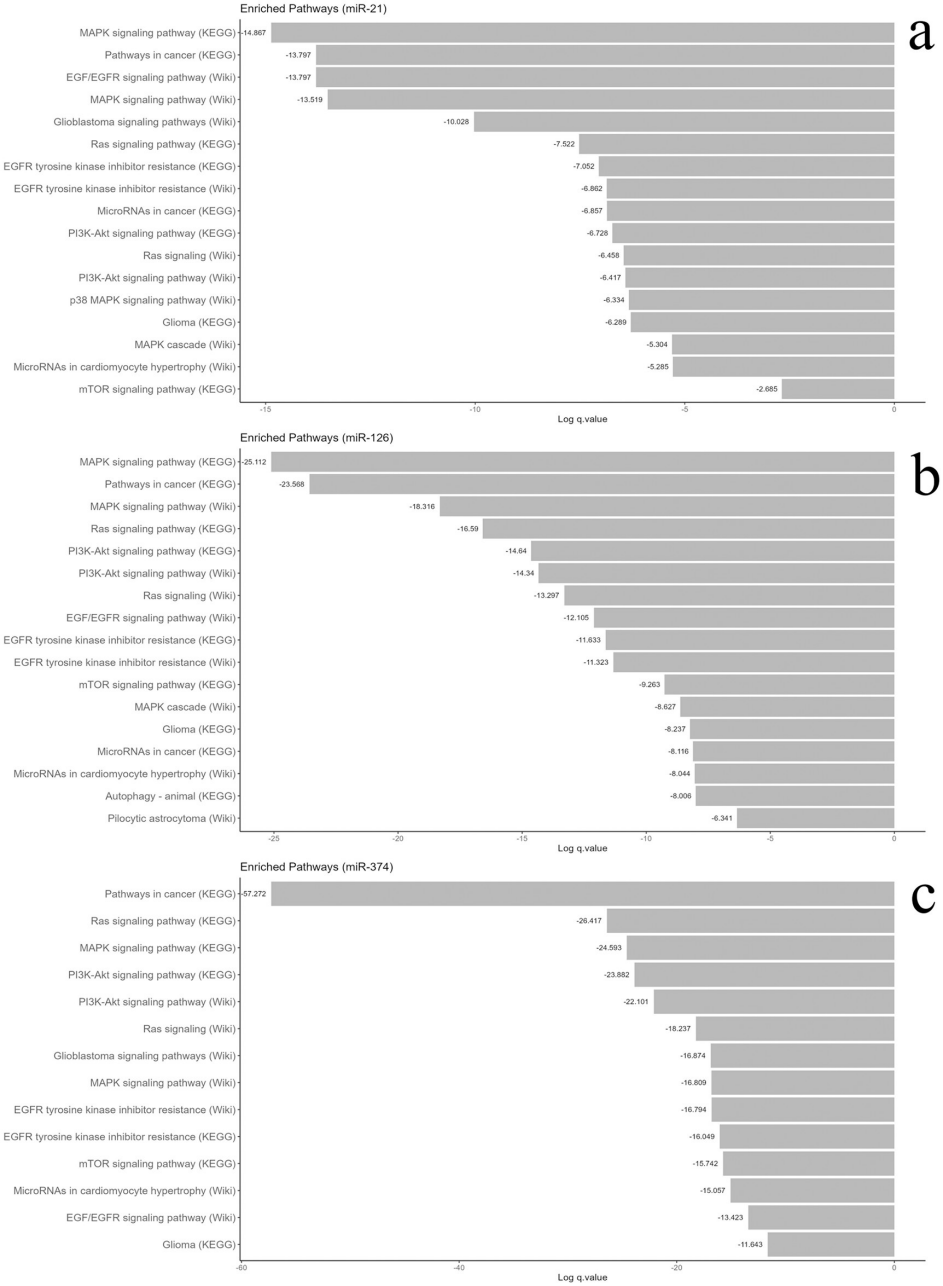
Functional enrichment of autophagy-related genes. Kyoto Encyclopedia of Genes and Genomes (KEGG) pathways and Wiki Pathways for **(a)**. miR-21, **(b)**. miR-126 and **(c)**. miR-374a.

In this study, we employed the MiRDB database (https://mirdb.org) [22,23] to identify target genes for miR-21, miR-374, and miR-126. We specifically selected miRs that exhibited statistical significance between HGG and LGG groups in our investigation and only included genes with a target score of 50 or above in MiRDB [24] (S1–S3 Files). The analysis revealed 154 genes associated with miR-21, 171 genes with miR-126, and 162 genes with miR-374a. To understand the biological processes involved, we conducted functional enrichment analysis using Metascape (http://metascape.org), focusing on pathways related to cancer, cell cycle, cell survival, and autophagy. The systematic exploration utilized the KEGG pathway (https://www.kegg.jp/en/) [25,26] WikiPathways, and oncogenic signature gene set enrichment analysis, with an adjusted p < 0.05 as the cutoff value [22]. For miR-21 targeted genes, the analysis uncovered significant involvement in 64 KEGG pathways, 83 Wiki Pathways, and 7 oncogenic signature gene sets. Notable pathways included MAPK signaling, cancer pathways, EGF/EGFR signaling, Glioblastoma signaling, Ras signaling, EGFR tyrosine kinase inhibitor resistance, miRs in cancer, PI3K-AKT signaling, p38 MAPK signaling, glioma, miRs in cardiomyocyte hypertrophy, and mTOR signaling (Fig 7A). Similarly, miR-126-targeted genes displayed significant involvement in 82 KEGG pathways, 89 Wiki Pathways, and relevant processes such as MAPK signaling, cancer pathways, Ras signaling, PI3K/AKT signaling, EGF/EGFR signaling, mTOR signaling, Glioma, miRs in cancer, miRs in cardiomyocyte hypertrophy, Autophagy, and Pilocytic astrocytoma (Fig 7B). Furthermore, miR-374a-targeted genes exhibited significant association with pathways such as cancer, Ras signaling, MAPK signaling, PI3K-AKT signaling, Glioblastoma signaling, EGFR tyrosine kinase inhibitor resistance, mTOR signaling, miRs in cardiomyocyte hypertrophy, EGF/EGFR signaling and glioma (Fig 7C). In addition to autophagy, we found the MAPK pathway as a common target for miR-21, miR-374, and miR-126, comprising subfamilies ERK, JNK/SAPKII, and p38 MAPK using functional enrichment analysis.

## Discussion

Our research, as the first of its kind from Pakistan, specifically examines the validation of autophagy-associated genes and MicroRNAs (miRs) about glioma prognosis within our population. We investigate the intricate relationship between autophagy and miRs, exploring how this interaction may influence the development and survival of glioma. The results of our study provide valuable insights into the demographic and clinical characteristics of glioma patients in our population. The most common histopathology groups observed were astrocytoma (36%) and glioblastoma (32%), followed by oligodendroglioma (12%). Among the 50 patients recruited, there was a male predominance, with 66% males and 34% females, and the median age was 35 years. The onset of glioma was notably higher in individuals above 40 years of age, indicating an early risk for glioma development, aligning with the findings of a study conducted by Thambi et al [27]. While both genders are equally susceptible to glioma, our study showed a lower likelihood of glioma prevalence in women compared to men. This observed trend may be associated with gender disparities in developing countries, where females are twice as likely to delay surgery due to financial constraints, travel difficulties, and decision-making processes [28,29].

The association analysis conducted in our study unveiled significant relationships between key prognostic markers Ki-67, IDH-1, ATRX and p53—and various clinical parameters encompassing age, histological type, treatment modalities, and tumor grade. Specifically, Ki-67 demonstrated significant correlations with age, histological type, radiotherapy, tumor grade, and current status. Notably, Ki-67, a widely accepted marker for cell proliferation in pathology, exhibited higher expression in high-grade glioma patients compared to low-grade ones. Interestingly, among adult glioma patients, Ki-67 expression was lower in those aged 40 and above, aligning with previous findings [30]. In line with prior research, our study highlighted the prevalence of IDH1 mutation in younger glioma patients, which also correlated with improved overall survival. Additionally, our data shed light on the association between IDH-1 wildtype status and high-grade gliomas. This reinforces the importance of IDH1 as a prognostic indicator in glioma management [31,32]. Furthermore, our findings indicated a correlation between mutant p53 status and chemotherapy. Consistent with previous studies, this suggests a potential link between p53 mutation and chemoresistance to TMZ in glioblastoma multiforme (GBM) [33]. This underscores the significance of p53 assessment in guiding therapeutic decisions and predicting treatment outcomes in glioma patients.

Subsequently, we investigated autophagy genes and associated microRNAs to discern their roles in glioma progression. Autophagy, initially recognized as a protective mechanism during metabolic stress, has garnered attention for its potential involvement in cancer promotion, metastasis, recurrence, and chemoresistance in advanced tumor stages [34,35]. This dual role underscores the complex and context-dependent nature of autophagy in cancer biology, prompting our exploration of its association with glioma. Utilizing RT-qPCR on fresh tissues, we sought to elucidate the involvement of autophagy in different glioma grades. In our analysis, LC3-II was utilized to assess autophagic flux, revealing pronounced punctate expression in both low and high-grade gliomas, although lacking statistically significant differences between the grades. While informative on autophagosome formation, this assay may not directly gauge autophagic flux [36]. Subsequently, employing RT-qPCR for LC3 quantification, we uncovered significantly elevated LC3 transcript expression in low-grade gliomas compared to high-grade ones [37]. Contrary to Aoki et al., who confirmed robust LC3 expression in glioblastoma tissues, contrasting with no LC3 staining in normal brain samples [38].

Furthermore, our study revealed a significant downregulation of ULK2 expression in high-grade gliomas compared to low-grade ones, consistent with findings by Shulka et al. This downregulation of ULK2 in glioblastoma aligns with reported promoter hypermethylation and ULK2 downregulation in normal brain tissue. The association of ULK1 and ULK2 as transcriptional targets of p53, crucial for sustained autophagy activity induced by DNA damage, further supports our results [39]. Considering the slower growth rate of low-grade gliomas compared to high-grade ones, it is reasonable to infer that nutrient stress is more pronounced in high-grade gliomas [40]. Consequently, we anticipate moderate mTOR activity and a higher autophagic rate in high-grade gliomas compared to low-grade ones. These findings align with previous studies indicating increased autophagic activity in high-grade gliomas [41,42].

Additionally, we evaluated the PI3K/AKT/mTOR pathway, focusing on the AKT gene, which exhibited significant upregulation in high-grade gliomas compared to low-grade ones, suggesting increased tumor proliferation in the malignant stage. However, PTEN and PI3K showed no significant changes between the groups, consistent with a study highlighting the essential role of the PI3K/AKT signaling pathway in glioma development and progression [43].

In this study, we investigated autophagy-associated miRNAs (miR-7, miR-21, miR-30, miR-100, miR-204, miR-126, and miR-374) and observed a significant upregulation of miR-21, downregulation of miR-126 and miR-374 in HGG tissues compared to LGG, in line with previous findings [44,45]. This study has identified that miR-374 is downregulated while AKT is upregulated in high-grade glioma supported by previous study [46]. Conversely, the tumor suppressor miR-126 was found to be downregulated in HGG. Previous research has demonstrated the anti-tumor effect of miR-126 in glioma, suggesting a potential inverse relationship between miR-126, PI3K, and AKT in glioma cell lines. miR-126 acts as a tumor suppressor by targeting, among others, AKT and PI3K [17,47].

Furthermore, correlation analysis using Spearman’s test identified a significant strong positive correlation between the expression of ULK2 and miR-7 in the LGG group and this correlation significantly reduced in HGG group as reported previously that miR-7 impairs autophagy. Previous studies have reported correlations between miR-7, PI3K/AKT, mTOR, and ULK2 in various cancers [48–50], but, to our knowledge, none have explored these correlations based on glioma gradesmiR-7 not only inhibits autophagy by directly repressing ULK2 but, also targets LKB1-AMPK-mTOR signaling pathway thus inducing cell proliferation [49]. However, in this study, we observed a decline of correlation between miR-374 and ULK2 from LGG to HGG. line with previous studies where high expression of miR-374 is associated with autophagy induction [51]. The intercorrelation among autophagy genes became more pronounced with increasing glioma grade in the present study, providing evidence for potential pathways of gliomagenesis involving the interplay of miR and autophagy markers. Notably, ULK2 and AKT exhibited a significant correlations with most autophagy markers in HGG, underscoring their pivotal roles as downstream targets of the PI3k-Akt signaling pathway [52]. PTEN correlated with ULK2 in LGG indicated its tumor suppressive role and its deficiency has been associated with promoting tumors indirectly through the dysregulation of PI3K/AKT [53,54].

We investigated the association of autophagy-associated mRNA and miRs with overall survival (OS) in glioma patients. The results demonstrated a significant association between the expression levels of ULK2, UVRAG and miR-374 with OS, indicating that low expression levels were linked to poor prognostic outcomes. However, miR-126 exhibited a significantly different expression pattern in OS, albeit with marginal significance. Our study confirms miR-21 as an independent prognostic marker in glioma, aligning with previous research [55]. In subgroup overall survival analyses based on well-established molecular markers of glioma (i.e., IDH, p53, Ki-67, ATRX), IDH wildtype and high Ki67 expression emerged as a significant predictor across all subgroups irrespective of autophagy markers. Studies have suggested that low expression of ULK2 might be associated with its promoter hypermethylation, causing autophagy by ULK1/ULK2 for glioma progression [39]. High miR-21 expression was associated with poor prognosis individually while observed in combination with IDH wildtype and high Ki67. Thus study confirms miR-21 as an independent prognostic marker in glioma, aligning with previous research [56]. Low expression of miR-374 was associated with poor overall survival individually as well as in subgroups of IDH wildtype and Ki67 high and P53 wildtype [46]. Similarly, low expression of the tumor suppressor miR-126, particularly in the context of high Ki67 and IDH wildtype, predicted a worse prognostic effect in our study. The inhibitory role of miR-126 induced autophagy against glioma survival, coupled with its promoter methylation, may contribute to tumor development [57,58].

Through functional enrichment analysis, we identified the following pathways MAPK, EGF/EGFR, Ras, EGFR, PI3K-AKT signaling, MAPK signaling, Glioblastoma signaling, pathways as a common target for miR-21, miR-374, and miR-126. Our study approach was to link and identify miRs direct and indirect targets in cellular signaling pathways with glioma, similar to the previous research on miR-196a-5p and its role in with MAPK signaling in glioma [59]. Our enrichment analysis highlights miR-21 target genes that were found to be associated with FGF1 and key tumor suppressor genes DAXX, TP53, TGFB2. Studies have suggested that miR-21 could mediate tumor promoting activities by impairing TP53 and its role in chemotherapy induce-DNA damage, making it chemoresistance and malignant. Likewise, the apoptotic mediator DAXX, is directly targeted by miR-21 and helps stabilize p53 and mediated TGFB apoptosis. It is negatively regulated by multiple components of miR-21 pathway making glioma resistant to TGF-β. The phenotypic effects observed of miR-21 downregulation are that of repression of p53, TGF-β and apoptotic pathways [60]. This interplay of PTEN inactivation, EGFR and AKT activation occurs frequently in GBM and is suppressed by inhibiting miR-21 [61]. The results of our enrichment analysis emphasize that miR-374 is associated with ErbB, mTOR, cell cycle signaling and tumorigenesis. Its predicted target genes include four hub genes, CCND1, SP1, CDK6 and CDK4, which are directly or indirectly involved in glioma development. CDK4 and CDK6 are two genes members of CDK family, their dysregulation could promote proliferation of GBM [46]. A study has also reported multiple roles of OLFML3 gene in malignant glioma grades 2 and 3, indicating its potential as a therapeutic target and prognostic marker [62].

MiR-126 has been identified as playing a complex role in glioblastoma multiforme (GBM), acting as both an oncomir and a tumor suppressor under different conditions. Studies have demonstrated that miR-126 targets negative regulators of the VEGF pathway, leading to increased VEGF signaling and subsequent angiogenesis. This effect is further amplified in the presence of bFGF, which also stimulates endothelial cell function and vascular network formation. The involvement of miR-126 in these pathways underscores its potential as a therapeutic target in GBM, where inhibiting its function could disrupt the tumor’s ability to sustain its blood supply and growth [63].

MiR-126 plays a critical role in glioblastoma by targeting and downregulating the expression of insulin receptor substrate-1 (IRS-1). This regulatory relationship between miR-126 and IRS-1 has significant implications for the behavior of glioma cells. Specifically, the upregulation of miR-126 leads to the suppression of IRS-1, which is associated with the promotion of glioma cancer stem cell formation. This suggests that miR-126 can contribute to the maintenance and proliferation of cancer stem cells in glioblastoma, thereby enhancing the tumor’s aggressiveness and resistance to conventional therapies [64]. Studies also suggest that this inverse regulation of miR-126 and IRS-1 could trigger glioma cancer stem cell formation. Emerging data also suggests that miR-126 could be a key modulator in preserving stemness capacity of cancer initiating cells. One of its important roles is in neurotrophin pathway involved neural cell differentiation, through modification of PI3K/Akt signalling. Another important target of miR-126 is KRAS, which is studied to be involved in cellular differentiation via PI3K/Akt pathway. miR-126 can also inhibit invitro proliferation, migration and promotes apoptosis of glioma by targeting the regulation of PTEN/PI3K/Akt and MDM2-p53 pathways. This mechanism suggests the tumor suppressive role of miR-126 [47,65]. Further, miR-126 may alter cellular mechanisms involved in cancer pathogenesis by suppressing translation of numerous validated target genes such as PI3K, KRAS, EGFL7, CRK, ADAM9, HOXA9, IRS-1, SOX-2, SLC7A5 *and* VEGF. These target genes and their role suggests miR-126 multi-functional role in angiogenesis, tumor growth and invasion [64].

In conclusion, our study significantly advances the understanding of glioma biology within the Pakistani population. By elucidating the tumor-suppressive role of autophagy in low-grade glioma and correlating the expression levels of key molecular markers with overall survival, we provide valuable insights into the prognosis and potential therapeutic targets for glioma patients. Our findings underscore the importance of population-specific factors in shaping survival outcomes, emphasizing the unique significance of miR-21 as an independent prognostic biomarker within this context. Furthermore, our research initiative lays the groundwork for the development of diagnostic and prognostic tools tailored to the Pakistani population. Through future investigations using cell culture-based techniques to manipulate miR expression, we aim to deepen our understanding of the intricate relationship between autophagy markers and miRs, potentially leading to the identification of novel therapeutic targets for glioma treatment. It is essential to acknowledge that similar observations have been reported by researchers globally, highlighting the collective effort in advancing glioma biology. Our study adds to this growing body of knowledge, contributing to the ongoing evolution of research on the complex interplay between autophagy, molecular markers, and patient outcomes across diverse demographic and geographic contexts. Overall, our findings underscore the importance of considering population-specific factors in understanding glioma pathogenesis and prognosis, ultimately guiding future therapeutic strategies.

## Supporting information

S1 Fig Receiver operating characteristics (ROC) for significant genes and miRs. (TIF)

S2 Fig Receiver operating characteristics (ROC) for molecular markers. (TIF)

S1 Table List of primers for autophagy associated genes and microRNAs used in the qPCR array. (XLSX)

S1 File Functional enrichment analysis of differentially expressed miR-374a target genes and pathways from miRDB. (XLSX)

S2 File Functional enrichment analysis of differentially expressed miR-126 target genes and pathways from miRDB. (XLSX)

S3 File Functional enrichment analysis of differentially expressed miR-21 target genes and pathways from miRDB. (XLSX)

S1 Data (XLSX)
