# Supplementary figures and images for: Autophagy-associated biomarkers ULK2, UVRAG, and miRNAs miR-21, miR-126, and miR-374: Prognostic significance in glioma patients
Source: PLoS One. 2024 Sep 30;19(9):e0311308. doi: 10.1371/journal.pone.0311308 (PMC11441661; doi:10.1371/journal.pone.0311308)

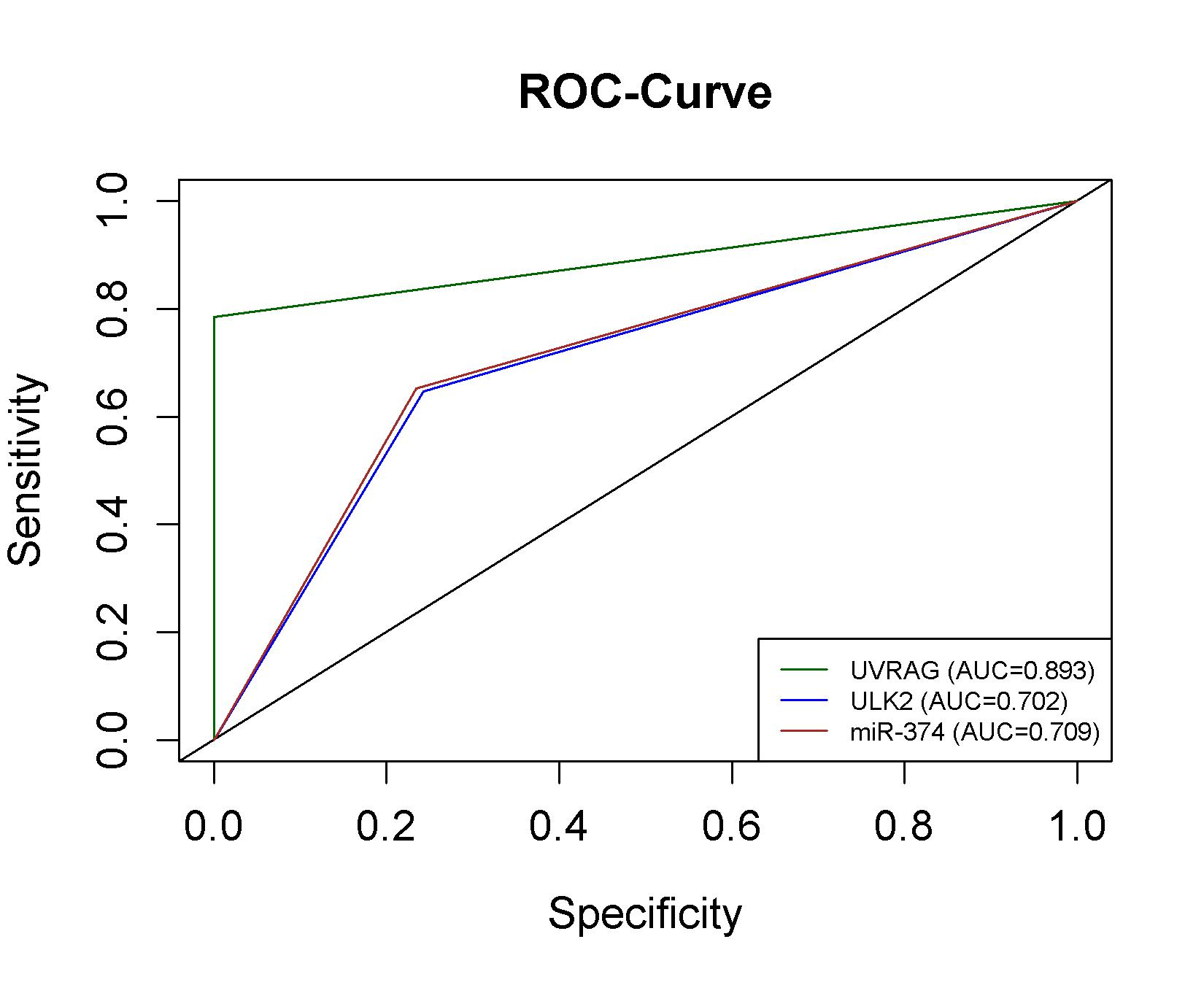

Supplement: S1 Fig — (TIF) [file pone.0311308.s001.tif]

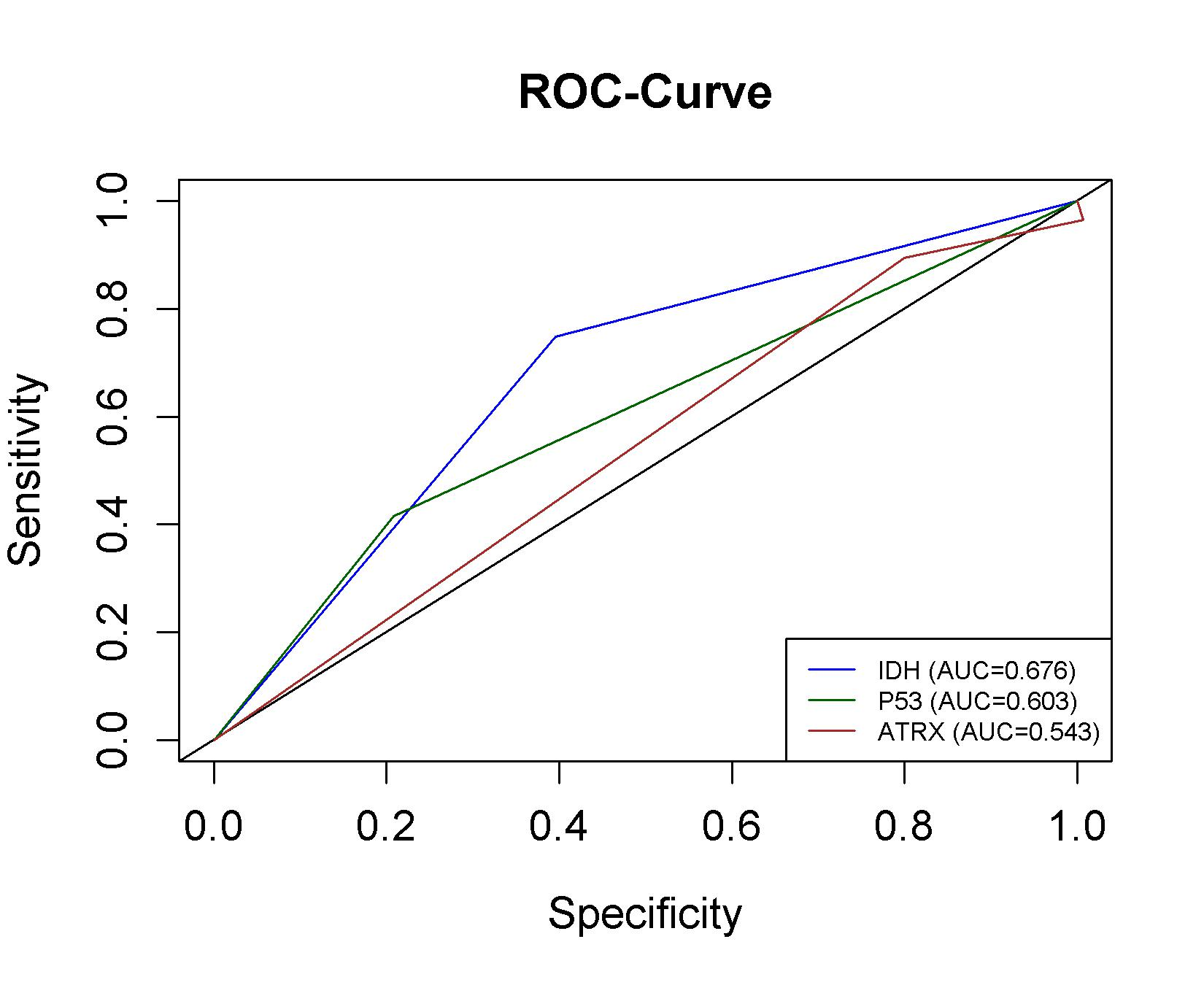

Supplement: S2 Fig — (TIF) [file pone.0311308.s002.tif]
